# Defect engineering for enhanced optical and photocatalytic properties of ZnS nanoparticles synthesized by hydrothermal method

**DOI:** 10.1038/s41598-023-43735-1

**Published:** 2023-10-05

**Authors:** E. Muhammed Jubeer, M. Aslam Manthrammel, P. A. Subha, Mohd Shkir, K. P. Biju, S. A. AlFaify

**Affiliations:** 1 Department of Physics, Farook College, University Of Calicut, Kozhikode, 673632 Kerala India; 2 Department of Physics, Faculty of Science, King Khalid University, P.O. Box-9004, 61413 Abha, Saudi Arabia; 3 Department of Physics, Govt. Arts and Science College, Kozhikode, Calicut, 673018 Kerala India

**Keywords:** Environmental sciences, Energy science and technology, Engineering, Materials science, Nanoscience and technology, Physics

## Abstract

Defect engineering is a promising method for improving light harvesting in photocatalytic materials like Zinc sulphide (ZnS). By altering the S/Zn molar ratio during hydrothermal processes, Zn and S defects are successfully introduced into the ZnS crystal. The band structures can be modified by adding defects to the crystal structure of ZnS samples. During the treatment process, defects are formed on the surface. XRD and Raman studies are used for the confirmation of the crystallinity and phase formation of the samples. Using an X-ray peak pattern assessment based on the Debye Scherer model, the Williamson-Hall model, and the size strain plot, it was possible to study the influence of crystal defect on the structural characteristics of ZnS nanoparticles. The band gap (E_g_) values were estimated using UV–Vis diffuse spectroscopy (UV–Vis DRS) and found that the E_g_ is reduced from 3.28 to 3.49 eV by altering the S/Zn molar ratio. Photoluminescence study (PL) shows these ZnS nanoparticles emit violet and blue radiations. In keeping with the results of XRD, TEM demonstrated the nanoscale of the prepared samples and exhibited a small agglomeration of homogenous nanoparticles. Scanning electron microscopy (SEM) was used to examine the surface morphology of the ZnS particles. Inductively Coupled Plasma Optical Emission Spectroscopy** (**ICP-OES) and X-ray photoelectron spectroscopy (XPS) were used to evaluate and validate the elemental composition. XPS results indicate the presence of defects on the prepared ZnS nanoparticles. For the investigation of vacancy-dependent catalytic activity under exposure to visible light, defective ZnS with different quantities of Zn and S voids are used as catalysts. The lowest S/Zn sample, ZnS0.67 and the highest S/Zn sample, ZnS3, show superior photocatalytic activity.

## Introduction

Zinc sulphide (ZnS) is a compound semiconductor with a wide direct band gap having n-type conductivity. It is considered a viable candidate for light-emitting diodes, electroluminescent devices, flat panel displays, infrared windows, sensors, lasers, and solar cells^1–3^. Numerous methods, including electrochemical deposition, microemulsion^4^, solvothermal^5^, sol–gel^6^, co-precipitation^7^, combustion synthesis^8^, pyrolysis, hydrothermal, laser ablation, and vapor deposition^9^, have been used to fabricate ZnS nanostructures. The hydrothermal method is adaptable, productive, and able to be adjusted; it doesn't require milling or calcination, has low contamination, and is cost-effective. It also has a high ability to regulate the nucleation process^5,10^.

Customizing the chromatic discharge of nanomaterials is crucial for their use in light-emitting screens, field emitters, lasers, sensors, and optoelectronic devices^11,12^. ZnS nanocrystals exhibit blue, green, and orange emissions^13,14^. The luminescence characteristics of ZnS particles have been altered by doping with various transition elements and rare-earth metals^15,16^. The optical characteristics are affected by defects, crystal structure, size, and shape. These studies show the ability to adjust several emission characteristics from pure ZnS nanocrystals with various defect features. Despite significant efforts to investigate the optical features, the sources of various photoluminescence (PL) bands from ZnS are infrequently addressed. The ZnS luminescence properties are typically attributed to surface states^17^, Sulphur vacancies^18^, Zn vacancies^19^, elemental Sulphur species, or impurities in ZnS^20^. There are many hanging bands and imperfections in the surface of ZnS due to its diverse interface topologies and larger specific surface areas^21^.

The global scarcity of freshwater supplies has long been ingrained in the public's eyes. The world is expected to be water-stressed by 2025^22^. Water pollution harms the ecosystem balance, so humans will be affected by clean water scarcity shortly. Water-related diseases include communicable diseases (waterborne, water-washed, water-based, and water-related vector-borne diseases) and noncommunicable diseases caused by chemically polluted water^23^. According to Hermabessiere et al., many plastics in the water are hazardous to a broad spectrum of organisms^24^. Several diseases are caused by chemical waste, including anemia, low blood platelets, headaches, cancer risk, and various skin problems^25^. Semiconductor photocatalysis is the most promising and successful approach for competing with water contaminants. ZnS is an important semiconductor photocatalyst in the II-VI group. ZnS is only sensitive to UV light absorption because of its broad bandgap energy. The development of visible-light-active photocatalysts capable of utilizing the greatest amount of solar light is an intriguing research area^26^. It is still challenging to improve visible photocatalytic activity by improving charge transfer and efficient charge separation^27^. The other hurdles in this field are low photocatalytic efficiency for visible-light photocatalysts, low mobility of charge carriers, inferior stability of photocatalyst^28^, high recombination rate of electron–hole pairs^29^ and cost-effectiveness at the commercial level^30,31^. The primary disadvantage of ZnS catalysts is their irreversible agglomeration during the photocatalytic processes and limited recyclability, which reduces the photocatalytic degradation efficiency^32^.

Defect engineering is another promising method for improving light harvesting in PC materials^33–36^. Semiconductor photocatalyst defects can function as adsorption sites for charge transfer that prevent the recombination of photoinduced charge and add new energy levels to narrow the band gap that creates visible-light activity. By creating additional energy levels to photoexcited charge carriers' electronic structure and characteristics, vacancy defects can significantly alter the PC activity of a photocatalyst^37^. Doping is also an effective strategy for inducing these defects. The visible light photocatalysis of ZnS has been reported to be improved by the addition of extrinsic metal elements such as Copper^38^, Nickel^39^, Cadmium^40^, or nonmetal elements Carbon and Nitrogen^41,42^. The inherent characteristics of materials, such as crystalline phases, defect states, exposed facets, etc., of semiconductor photocatalysts are crucial for superior photocatalytic (PC) activities^37,43^.

This study presents a simple hydrothermal method for introducing S and Zn vacancies into the ZnS structure by changing the S/Zn molar ratios. Investigations are made into how vacancy-related features affect the photoluminescence and PC activity of ZnS in visible light.

## Experimental

### Synthesis

The nanoparticles (NP) of ZnS were prepared by the hydrothermal method, and the brief synthesis procedure followed. The required amount of ZnCl_2_ and SC(NH_2_)_2_ powder were dissolved in de-ionized water separately, and five drops of HCl were added to the ZnCl_2_ solution and stirred at room temperature for 1 h. Thiourea solution is then dripped into ZnCl_2_ solution, and the mixture is stirred at room temperature for another 1 h. 50 ml of the clear mixture was then charged to a Teflon-coated SS autoclave of 100 ml volume. The closed autoclave was placed in a furnace at a temperature of 220 °C for 12 h and then allowed to cool to room temperature. The nanoparticles in the solution were filtered and washed several times using de-ionized water to remove impurities in the sample. The final whitish product was dried at 60 °C for 1 h. Five samples were prepared by varying [S]/[Zn] molar ratios using the same procedure. Samples with [S]/[Zn] molar ratios of 0.66, 1, 1.5, 2, and 3 were prepared and named ZnS0.67, ZnS1, ZnS1.5, ZnS2, and ZnS3 for further characterization.

### Measurements

The structural studies of prepared samples were carried out by the X-ray diffractometer (XRD) using Rigaku Miniflex 600 X-ray diffractometer with Cu Kα (λ = 1.542 Å) radiation, operating at 15 mA and 40 kV. Cu target and graphite monochromator were used, and data was recorded in continuous scan mode from 100 to 800, with a step size of 0.020 at scan speed of 100 per minute. The vibrational study was carried out for all present samples using the Horiba Lab Ram HR Evolution Confocal Raman Spectrometer. The Raman spectrometer was operated using 532 nm (Diode Pumped Solid State Laser) at room temperature. X-ray photoelectron spectroscopy (XPS) of the ZnS nanoparticles was characterized by a Thermo-scientific NEXA Surface analyzer. The determination of the element content was carried out using a Thermo-scientific Icap 6300 ICP-OES. The Photoluminescence study was also carried out using Agilent Cary fluorescent spectrophotometer. Optical studies of all ZnS nanostructures were analyzed using The Cary 5000 UV–Vis-NIR spectrophotometer, in the wavelength range of 200–1200 nm at room temperature. The morphological studies of all the synthesized ZnS nanostructures were done using a ZEISS Gemini SEM 300. TEM and EDAX studies by using Tecnai G2 F20, FEI Company operating at 200 kV and OXFORD X- Max, respectively. To conduct a PC study, 0.03 g of ZnS photocatalyst is added to 25 ml of Methylene Blue (MB) solution. To prepare the MB dye solution, 10 mg of MB was added to 1L of distilled water, resulting in photocatalysts. The mixture was magnetically stirred for 1 h in darkness to attain adsorption–desorption equilibrium between the MB dye and catalyst. The PC studies were conducted from 11 a.m. to 2 p.m. on sunny days^39^. The solar intensity was measured in each hour during the experiment using a VAR TECH V6610 Digital Lux meter, and the intensity values were 56 k(lux), 79 k(lux), 62 k(lux), and 48 k (lux), respectively. The specific surface area of the samples is estimated through the Brunauer–Emmett–Teller (BET) method using BELCAT-M.

#### X-ray diffraction analysis (XRD)

**Figure 1:**
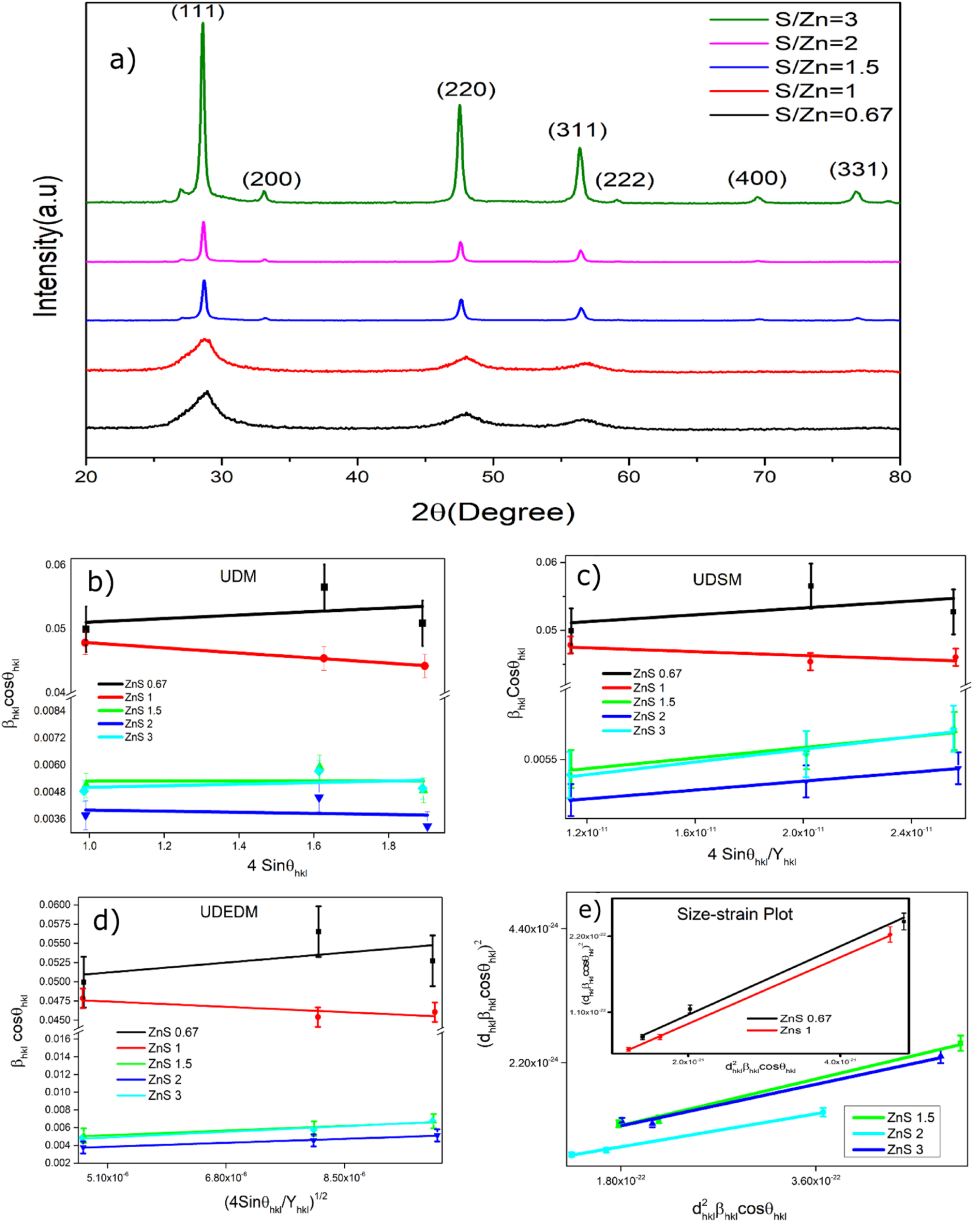
(**a**) XRD pattern of present samples. (**b**) UDM, (**c**) UDSM, (**d**) UDEDM, (**e**) size-strain plot of present samples.

The XRD spectra of the ZnS samples are depicted in Fig. 1a. Every one of the recognizable peaks could be ordered as the cubic ZnS in the standard reference information (ICDD 01-077-2100). Different lattice parameters such as interplanar spacing, unit cell volume, and lattice constant were determined from the Bragg condition and the Lattice Geometry conditions^44^ of the form:

1 $$2{d}_{hkl}sin \theta =\lambda$$

2 $$V={a}^{3}$$

3 $$d=\frac{a}{{(h}^{2}+{k}^{2}+{l}^{2}{)}^\frac{1}{2}}$$

where' λ 'is the wavelength of the X-ray used, which is 1.54056A°, (hkl) is the miller index of the crystal plane, and θ is Bragg's angle^45^. The lattice parameters of these ZnS powders synthesized using the hydrothermal method were determined utilizing the crystallographic planes relating to the Miller index (111), (220) and (311), which are consistent and in remarkable concurrence with the standard reference data 5.39 nm. A similar finding was reported by Quynh Hoa et al., well.^46^. The ZnS nanoparticle crystallite size, D, was determined utilizing the standard Scherrer's equation

4 $$D=\frac{N\lambda }{\beta cos\theta }$$

where β is full-width half maxima, and N is the crystallite shape factor^47^. A gaussian fitting with a Chi-square value of more than 0.98 was used in our analysis to determine the peak width. The current analysis used the line broadening of Si single crystal as a reference material to rule out the impact of the instrument. The crystallite size measured by substituting relevant information from the XRD is 2.8 nm, 2.9 nm, 27.2 nm, 36.7 nm, and 28.6 nm for the samples ZnS0.67, ZnS1, ZnS1.5, ZnS2, and ZnS3 respectively, from the peak corresponding to (111) plane. For lower S/Zn samples ZnS0.67 and ZnS1, the XRD spectrum shows broad peaks, which indicate the formation of quantum dots like ZnS particles. The crystallite size is more significant when the S/Zn molar ratio exceeds 1. Still, the high intensity of the peaks shows a higher crystallinity nature of the samples with a high S/Zn Molar ratio, M. Thambidurai et al*.,* also reported the same observation^48^. The formation of a little amount of ZnS in the wurtzite structure might also be the cause of the exceptionally weak diffraction signal at 26.93° for S rich sample (#01-075-1547)^20^. The peak positions and intensities are listed in Table 1.

**Table 1:** Structural parameters for ZnS NPs.

Sample	2θ (°)	Intensity (a.u)	Crystallite size (nm)	Interplanar spacing d_111_ (A°)		Lattice parameter a (A°)		Grain size from SEM (nm)
ZnS0.67	28.68	3945	2.8	3.11	Standard d_111_ = 3.12	5.39	Standard Value = 5.41	25.8
ZnS1	28.60	3543	2.9	3.12		5.40		57.7
ZnS1.5	28.69	32,265	27.2	3.11		5.38		93.9
ZnS2	28.64	21,354	36.7	3.11		5.39		158
ZnS3	28.51	18,084	28.6	3.12		5.42		85.4

##### Williamson–Hall (W–H) method

W–H models, for example, a uniform deformation model (UDM) could gauge the lattice strain, which was assessed in the ZnS NP due to the deformation of the grid. Different models, such as the Uniform Deformation Stress Model (UDSM), recognize the stress–strain connection. In contrast, the Uniform Deformation Energy Density Model (UDEDM) assesses the strain and is widely used for finding energy density^49,50^. We have focused on the three prominent peaks in the Williamson-Hall analysis and the other models, which correspond to the (111), (220), and (311) crystallographic planes.

##### Unified deformation model (UDM)

According to W–H method, the peak widening is owing to the crystal size and the impact of the strain. The influence of the strain is represented by the Eq. (5)

5 $${\beta }_{strain}=4\varepsilon tan\theta$$

where, $\varepsilon$ is the internal strain.

Hence, the total peak width is shown in Eq. (6) represent UDM^51^;

6 $${\beta }_{hkl}=4\varepsilon tan\theta +\frac{N\lambda }{Dcos\theta }$$

The UDM plot drawn 4 sin θ along the X-axis and β_hkl_ cos θ along the Y-axis of the prepared samples are shown in Fig. 1b.

The strain was assessed from the slop of linearly fitted diagrams and normal crystallite size from the Y-intercept^52^. The strain of ZnS nanoparticles is viewed as 2.7 × 10^–3^, − 3.9 × 10^–3^, 0.01 × 10^–3^, -0.25 × 10^–3^, 0.35 × 10^–3^ for ZnS0.67, ZnS1, ZnS1.5, ZnS2 and ZnS3 samples respectively. The negative slope of Williamson and Hall plots for the unit S/Zn molar proportion demonstrates the presence of compressive strain, which could initiate the negative strain mutilation. Nonetheless, the positive slope could be ascribed to the tensile strain or inside stress in the crystal owing to the thermal expansion during the crystal development^53^. In this manner, grid strain differs from a positive to a negative worth, explicitly relying upon the S/Zn molar proportion. The crystallite size is obtained as 2.9 nm, 2.7 nm, 26.2 nm, 32.6 nm, and 29.7 nm for the ZnS0.67, ZnS1, ZnS1.5, ZnS2, and ZnS3 respectively.

##### Uniform deformation stress model (UDSM)

The postulation of homogeneity and isotropy is not satisfied in all cases. To consolidate more sensible circumstances, an anisotropic methodology is taken on; consequently, the W–H condition is altered by an anisotropic strain ϵ. In USDM, the lattice deformation stress is considered uniform in all crystallographic orientations, expecting a little microstrain in the particles.

7 $$\mathrm{According to Hooks Law }\epsilon =\frac{\sigma }{{Y}_{hkl}}$$

where ϵ is the anisotropic strain and ${Y}_{hkl}$ is Young's modulus in the plane (h k l), and σ is the stress of the crystal.

In this approach, Eq. (6) is modified as follows^54^

8 $${\beta }_{hkl}Cos {\theta }_{hkl}=\frac{k\lambda }{D}+\frac{4\mathrm{\sigma sin}{\theta }_{hkl}}{{Y}_{hkl}}$$

Equation (8) is called the Uniform Deformation Stress Model (UDSM). Figure 1c shows the UDSM plot of the prepared samples with different S/Zn molar ratios. The slope of the fitted plot will give uniform stress, and the particle size can be evaluated from the Y-intercept^55^. The particle sizes are obtained as 2.9 nm, 2.8 nm, 36.7 nm, 51.4 nm, and 42.7 nm for ZnS0.67, ZnS1, ZnS1.5, ZnS2, and ZnS3, respectively. The microstrain values of different crystal planes of prepared samples are shown in Table 2.

**Table 2:** Structural parameters for ZnS NPs determined using different approaches.

Samples	Parameter	Scherrer	UDM	UDSM	UDEDM	SSP
ZnS 0.67	D (nm)	2.8	2.9	2.9	2.9	2.8
	ε (10^–3^)	50.4	2.7	(110) = 2.92 (220) = 3.15 (311) = 3.42	(110) = 3.66 (220) = 3.80 (311) = 3.96	51.2
	σ (G Pa)			0.25	0.3	
	U (kJm^−3^)				582.34	
ZnS 1	D (nm)	2.9	2.7	2.8	2.8	2.9
	ε (10^–3^)	48.4	−3.97	(110) = −1.63 (220) = −1.76 (311) = −1.91	(110) = 1.96 (220) = 2.04 (311) = 2.12	36.6
	σ (G Pa)			−0.15	0.16	
	U (kJ m^−3^)				167.513	
ZnS 1.5	D (nm)	27.2	26.3	36.8	38.9	32.7
	ε (10^–3^)	5.15	0.01	(110) = 1.29 (220) = 1.40 (311) = 1.52	(110) = 1.49 (220) = 1.55 (311) = 1.62	14.4
	σ (G Pa)			0.11	0.13	
	U (kJ m^−3^)				974.52	
ZnS 2	D (nm)	36.7	32.6	51.4	55.2	46.0
	ε (10^–3^)	3.8	−0.025	(110) = 1.09 (220) = 1.17 (311) = 1.27	(110) = 1.26 (220) = 1.31 (311) = 1.36	10.5
	σ (G Pa)			0.09	0.11	
	U (kJ m^−3^)				693.5	
ZnS 3	D (nm)	28.6	29.7	42.7	46.2	36.6
	ε (10^–3^)	4.9	0.35	(110) = 1.55 (220) = 1.68 (311) = 1.82	(110) = 1.78 (220) = 1.86 (311) = 1.93	13.8
	σ (G Pa)			0.01	0.15	
	U (kJ m^−3^)				138.997	

##### Uniform deformation energy density model (UDEDM)

Equation (6) is changed to the structure where energy per unit volume (energy density), 'u' is considered. As per Hooke's law, the 'u' as a strain component is u = ε^2^Y_hkl_/2. In the strain–stress connection, all the proportionality constants become, at this point, not independent when the strain energy density is considered^56^. Thus, the above equation can be written as

9 $${\beta }_{hkl}Cos {\theta }_{hkl}=\frac{k\lambda }{D}+\frac{4\mathrm{sin}{\theta }_{hkl}{(2u)}^\frac{1}{2}}{({Y}_{hkl}{)}^\frac{1}{2}}$$

The plot, ${\beta }_{hkl}Cos {\theta }_{hkl}$ Vs $\frac{4\mathrm{sin}{\theta }_{hkl}{2}^\frac{1}{2}}{({Y}_{hkl}{)}^\frac{1}{2}}$ is the UDEDM plot^57^ of the prepared samples with different S/Zn Molar ratios, shown in Fig. 1d. The Y-intercept gives the average crystallite size, and the values are 2.9 nm, 2.8 nm, 38.9 nm, 55.2 nm, and 46.2 nm for ZnS0.67, ZnS1, ZnS1.5, ZnS2 and ZnS3 respectively. The energy density values evaluated from the slopes are listed in Table 2.

##### Size-strain plot (SSP)

The Williamson-Hall plots reveal that the line widening is fundamentally isotropic. This underscores that the diffracting areas are isotropic because of the strain. In instances of isotropic line expansion, a superior assessment of the size-strain boundaries can be obtained by taking into account a SSP. This technique has the advantage that less significance is given to information from reflections at high angles with lower accuracy. In this technique, it is accepted that the 'strain profile' is explained by a Gaussian function and the 'crystallite size' profile by a Lorentzian function^58^, which is given by,

10 $${(d}_{hkl}{\upbeta }_{hkl}\mathrm{cos\theta }{)}^{2} =\frac{K}{D} ({d}_{hkl}^{2}{\upbeta }_{hkl}\mathrm{cos\theta })+( \frac{\varepsilon }{2}{ )}^{2}$$

where k is the shape factor, and D is the crystallite size. The plot $({d}_{hkl}^{2}{\upbeta }_{hkl}\mathrm{cos\theta })$ along the X-axis and ${(d}_{hkl}{\upbeta }_{hkl}\mathrm{cos\theta }{)}^{2}$ along the Y-axis, as shown in Fig. 1e is the Size-Strain Plot^59^. The slope of the fitted plot gives the crystallite size, and the Y-intercept helps to determine the strain. The calculated strain values for samples ZnS0.67, ZnS1, ZnS1.5, ZnS2 and ZnS3 were 51.2, 36.6, 14.4, 10.5, and 13.8, respectively. The values are listed in Table 2.

The crystallite size of these samples was measured using the Scherrer formula and other methods such as UDM, UDSM, UDEDM, and Size-Strain Plot (SSP). When different methodologies for estimating crystallite size were evaluated, it was noticed that the SSP model produced more accurate findings than the UDM, UDSM, and UDEDM methods. The assessment of the plots revealed that the data were more precisely matched using the SSP model. These results indicate that the SSP technique is a valid method for assessing crystallite size in ZnS samples with varying S/Zn ratios. The accuracy of the SSP model can be ascribed to its capacity to account for lattice strain and give a more thorough understanding of the crystal structure. Overall, this research highlights the need to employ appropriate methodologies for assessing crystallite size in nanocrystals^60,61^. The various models used to estimate strain in nanoparticles generated vary strain values, indicating that strain is not direction-dependent. This finding is crucial because it sheds light on the structural properties of nanoparticles as well as their behavior under various conditions^54^.

#### Raman analysis

**Figure 2:**
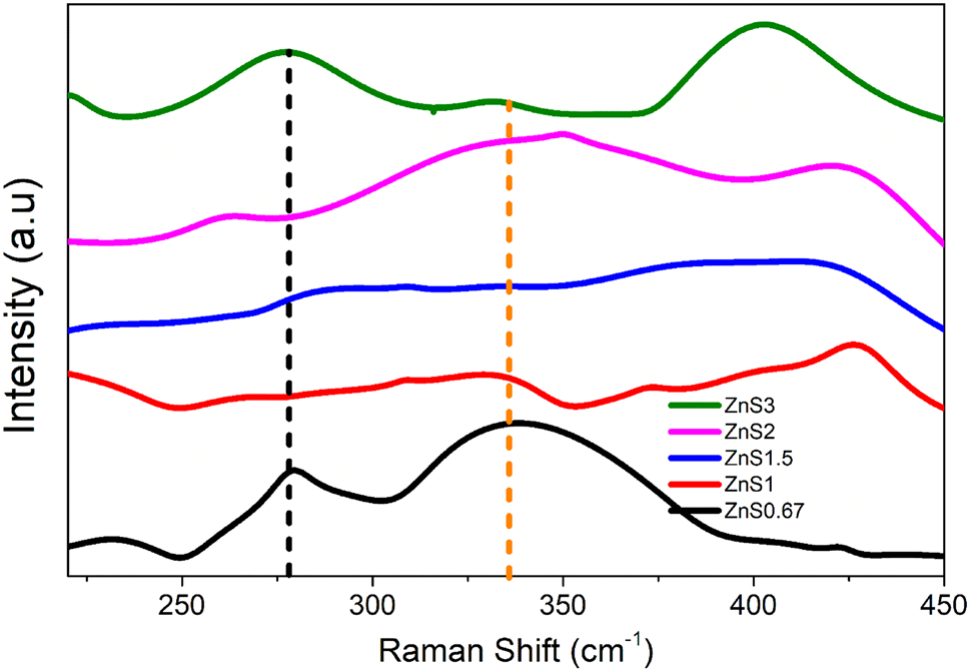
Vibrational Raman spectra of ZnS.

Through phonon interactions, changes in surface features can be inferred using the Raman vibrational investigation. The Raman Vibration Modes of the present samples are shown in Fig. 2. As Cheng et al*.,* reported, the only permitted modes at the zone center for the cubic ZnS phase are the longitudinal optical (LO) modes and transverse optical (TO) modes^62^. All samples showed a TO peak mode at around 270 cm^−1^ and LO peak at around 340 cm^−1^, supporting the cubic phase of ZnS. Notably, for both fundamental modes, there is a peak shift towards the shorter wavelength as the Zn vacancy (V_Zn_) increases to a certain level and then shifts to a higher wavelength. However, due to surface modulation, it is feasible to observe some other Raman modes at the nanoscale. The overtones of transverse acoustic phonons^63^ for all samples except ZnS2 were identified as the sources of the peaks around 235 cm^−1^. The Raman modes around 427 cm^−1^ for all samples except ZnS3 are due to TO + LA. The identical Raman modes were detected by Nilsen^64^, or due to the LO + TA vibrational modes as well as, which is in line with reports by S. Dhara et al*.,* and Zhiyuan Ye et al.^65,66^. The slight shift in the peaks may be due to the nanoconfinement effect. The other Raman vibration mode that appeared common for all samples is TO + TA mode at 390 cm^−1^^67^. Raman spectra revealed further information about the lattice defects. Along with the above-mentioned Raman vibration modes, some samples also exhibit defect-induced Raman modes. The samples ZnS0.67, ZnS1, ZnS1.5, and ZnS3 centered at 320 cm^−1^ have a lattice defect-induced phonon mode at X-W-L, as reported by Jun Zhou et al.^63^. The cubic crystalline phases are confirmed by Raman analysis, which is in good agreement with the XRD results.

#### Scanning electron microscope (SEM)

**Figure 3:**
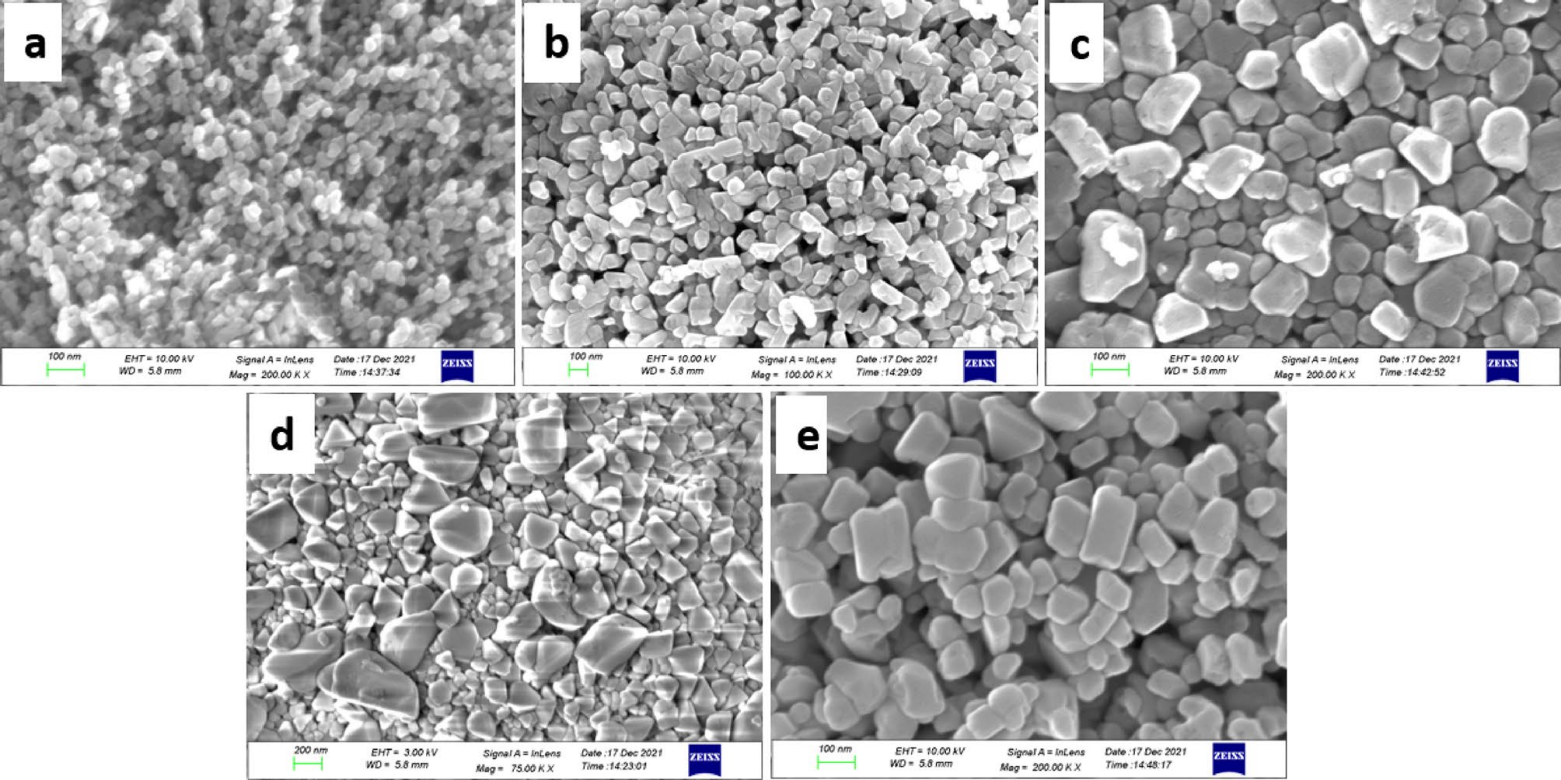
SEM image of (**a**) ZnS0.67, (**b**) ZnS1, (**c**) ZnS1.5, (**d**) ZnS2, (**e**) ZnS3.

The morphological study of the ZnS sample synthesized by the hydrothermal method uses SEM images shown in Fig. 3. It is obvious that the combination of grains is visible with uniform polygonal-shaped particles. The particles are shaped with a compact morphology and thickly stuffed. Normal grain sizes of the particles were found to be greater than the crystallite size determined from XRD, and the values are 25.8 nm, 57.7 nm, 93.9 nm, 158 nm, and 85.4 nm for ZnS0.67, ZnS1, ZnS1.5, ZnS2 and ZnS3 respectively. It is to be noted that the grain sizes assessed by XRD and SEM were exceptionally interesting. In SEM, the grain size was assessed by the differentiation between the clear grain boundaries, while in XRD, the estimation extended to the crystalline region that diffracted X-ray beams coherently. As needed, the XRD assessments provoked a smaller size^68^. The size distribution relationship with the S/Zn molar ratio is listed in Table 1.

#### Transmission electron microscopy (TEM) and energy-dispersive X-ray analysis (EDAX)

**Figure 4:**
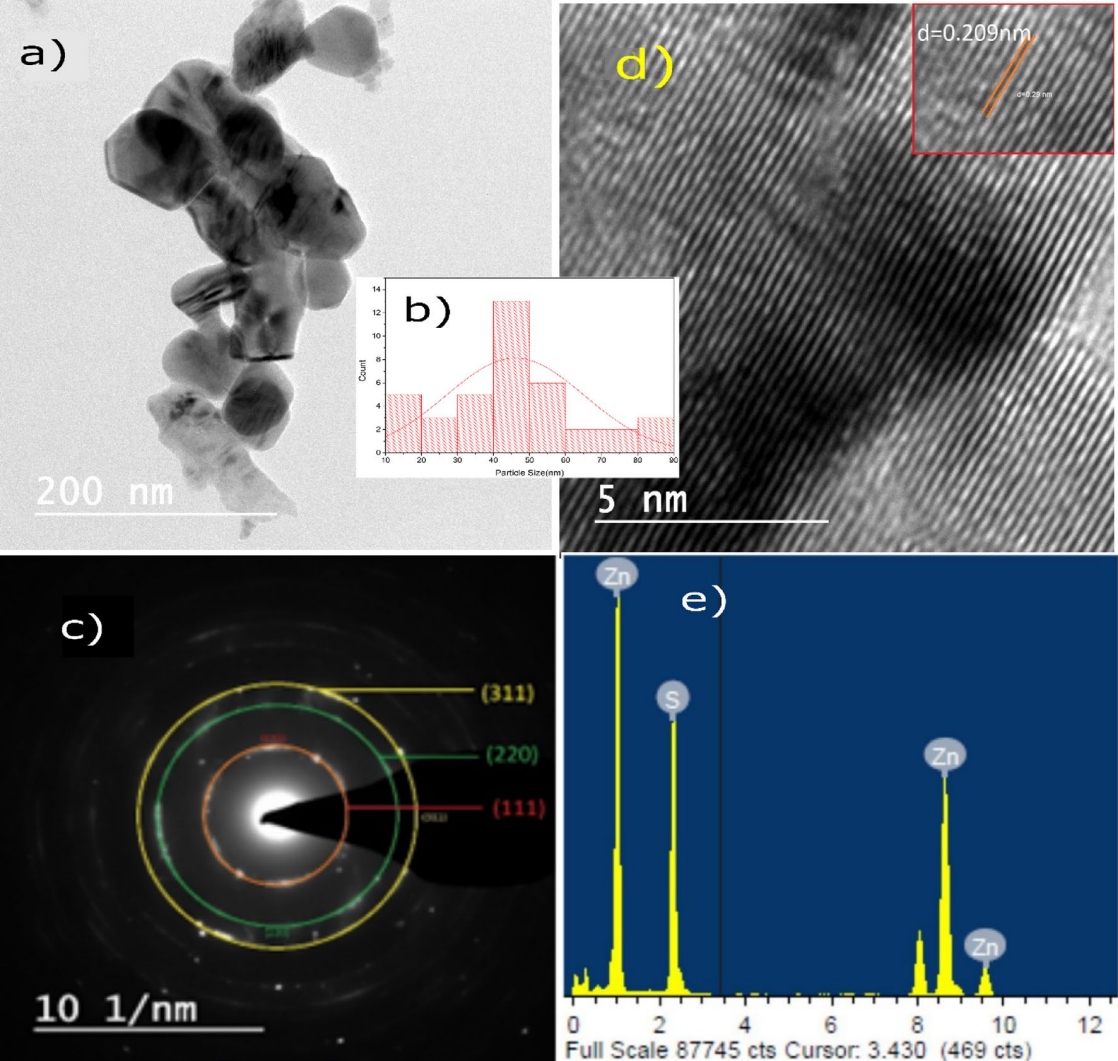
(**a**) TEM image of ZnS1. (**b**) Size distribution curve of ZnS1. (**c**) SAED pattern of ZnS1. (**d**) HRTEM image of ZnS1. (**e**) EDAX Spectrum of ZnS1.

The example with ZnS1 was additionally described by TEM (Fig. 4a) procedure joined by selected area electronic diffraction (SAED) in Fig. 4c. The TEM picture shows that, not every one of the particles is spherical. At the same time, any additional different shapes are scarcely noticed. The normal size of the particles assessed from the TEM images is 46.5 nm in the range of size 15.7–87.3 nm. The particle sizes derived by TEM images are obviously bigger than those from XRD. This indicates that the synthesis route produces ZnS agglomerate nanoparticles or can also be interpreted as XRD expecting normal atomic distribution in the examples. However, TEM gives the standard size of the particles, including the non-crystalline part^69^. From the SAED example of samples, it was seen that ZnS nanoparticles were polycrystalline, and the (111), (220), and (311) planes from the inside ring to the beyond SAED investigation affirm the Zinc blende structure of items, which is consistent with XRD and RAMAN results. A delegate HRTEM picture enlarging a round part of the structure is given in Fig. 4d. The interplanar distances of the crystal fringes are around 0.209 nm. The size distribution curve of ZnS1 is shown in Fig. 4b.

Typical EDAX analysis was performed to assess the constituents of the synthesized products, and the results are presented in Fig. 4e. Zn and S have produced four distinct signals demonstrating that the product was pure ZnS.

#### Inductively coupled plasma optical emission spectroscopy (ICP-OES)

Determining the lighter elements or volatile components in semiconducting materials can be difficult regardless of the analytical approach. High-energy photons, electrons, or ions impact the material during the measurement and may expel lighter or volatile components, damage the surface, or modify the oxidation state and crystal structures. ICP-OES (inductively coupled plasma optical emission spectroscopy) is a technology used for elemental analysis. Environmental analysis, geology, pharmaceuticals, and materials research are just a few sectors that use it extensively.

The ICP-OES data reveal that all samples follow the predicted S/Zn ratio trend (Table 3). The changes in S/Zn ratios observed by ICP-OES followed the predicted pattern of partial loss of volatile S species during ICP-OES sample preparation^70^. When exposed to acid, semiconductors that generate volatile hydrides, such as H_2_S, are vulnerable to elemental loss during digestion^70^. ICP-OES has different detection limits for each element and may be less sensitive than other methods for some elements or at low concentrations. If the concentration of Zn and S in the ZnS nanoparticles is less than the ICP-OES instrument's detection limit, this might explain the lower observed percentage. Moreover, Yang et al*.,* reported that the ICP-based overall analysis is not accurate for the S analysis^71^.

**Table 3:** Band Gap energy from DRS, Compositional for ZnS NPs from ICP-OES and BET analysis result.

Sample	E_g_ (eV)	ICP-OES (atomic %)			BET	Photodegradation efficiency (%)
		Sulphur	Zinc	S/Zn	S.A (m^2^ g^−1^)	
ZnS0.67	3.46	17.78	82.21	0.22	16.77	83.78
ZnS1	3.32	27.66	72.34	0.38	5.57	64
ZnS1.5	3.30	28.77	71.22	0.40	14.233	61.5
ZnS2	3.28	42.19	57.8	0.73	10.652	72
ZnS3	3.49	65.48	34.52	1.89	10.534	82

#### X-ray photoelectron spectroscopy (XPS)

**Figure 5:**
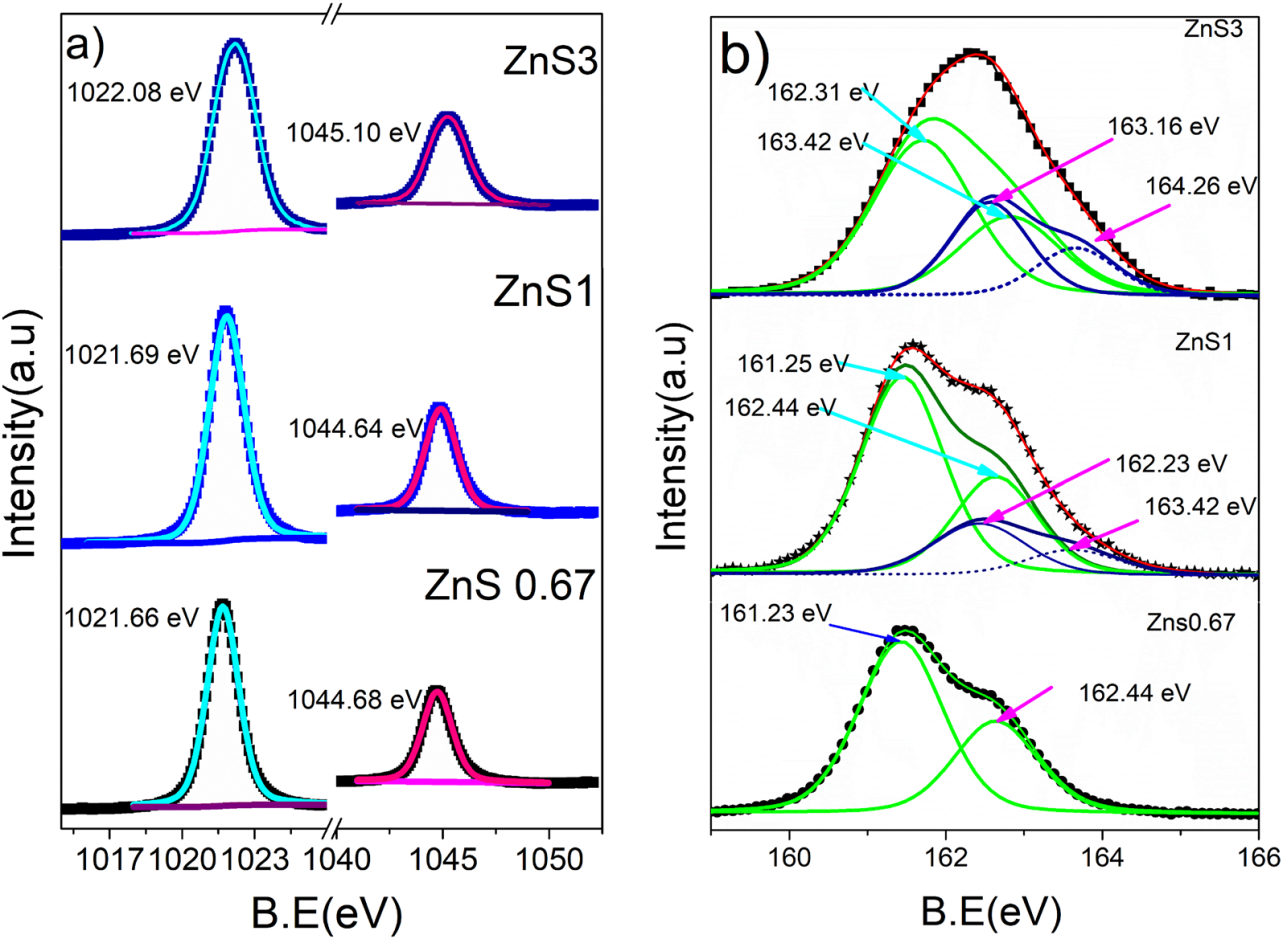
XPS spectra of ZnS0.67, ZnS1 and ZnS3 (**a**) Zn2P region XPS spectra (**b**) S2P region XPS Spectra.

X-ray photoelectron spectroscopy (XPS) is used to investigate the surface composition and chemical state. All binding energy (B.E) values in the XPS spectra were calibrated using the C 1 s line at 284.8 eV. Measurements were made of the XPS spectra of ZnS0.67, ZnS2, and ZnS3. The high-resolution XPS spectra (Fig. 5a) of Zn 2p for ZnS0.67 show two peaks at 1044.48 eV and 1021.26 eV, separated by around 23 eV. Goudarzi et al., and Z. Ye et al., stated that these are ascribed to Zn 2p_1/2_ and Zn 2p_3/2_^65,72^. Zn 2p_1/2_ and Zn 2p_3/2_ peaks of ZnS2 are detected at B.E values of 1021.69 eV and 1044.64 eV, respectively, while the same for ZnS3 is observed at 1022.68 eV and 1045.7 eV. As the S concentration rises, these peaks move slightly to higher B.E. Excess S atoms in S-rich ZnS can create extra chemical bonds or defects in the crystal. These modifications may result in greater binding energy for the Zn 2p electrons in XPS.

The S 2p_3/2_ and S 2p_1/2_ peaks for ZnS0.67 are positioned at around 161.23 eV and 162.43 eV, X. Yang et al., also reported the same finding^71^, respectively, which are typical ZnS features, however when the S content rises, the of the asymmetric core level XPS spectra of S 2p may be resolved into four peaks (Fig. 5b). For ZnS1, deconvoluted peaks of core level XPS spectra of S 2p are detected at 161.25 eV, 162.44 eV, 162.23 eV, and 163.42 eV. The B.E 161.25 eV and 162.44 eV are derived from S 2p_3/2_ and S 2p _1/2_ of S atoms bound in ZnS, whereas 162.23 eV and 163.42 eV are derived from the defect state in ZnS^37^. The defect percentage calculated from the area under the respective peaks in XPS is 18% for Zns1.

Deconvoluted peaks belonging to core level XPS spectra of S 2p of bonded S are seen at 162.31 eV and 163.42 eV, 163.16 eV, and 164.26 eV for sample ZnS3 (Fig. 5b). The B.E 162.31 eV and 163.42 eV are derived from S 2p_3/2_ and S 2p _1/2_ of S atoms bound in ZnS, while peaks relating to defects are observed at 163.16 eV and 164.26 eV. The area of the peaks corresponding to the defects is greater in Zns3, with 34% of the defects computed for the same. The XPS examination result shows that the defects are in the prepared samples. This implies that the concentration of defects may be regulated to some extent by adjusting the molar ratio of precursors utilized. X. Hao et al*.,* used the same method in their study^37^.

#### UV

**Figure 6:**
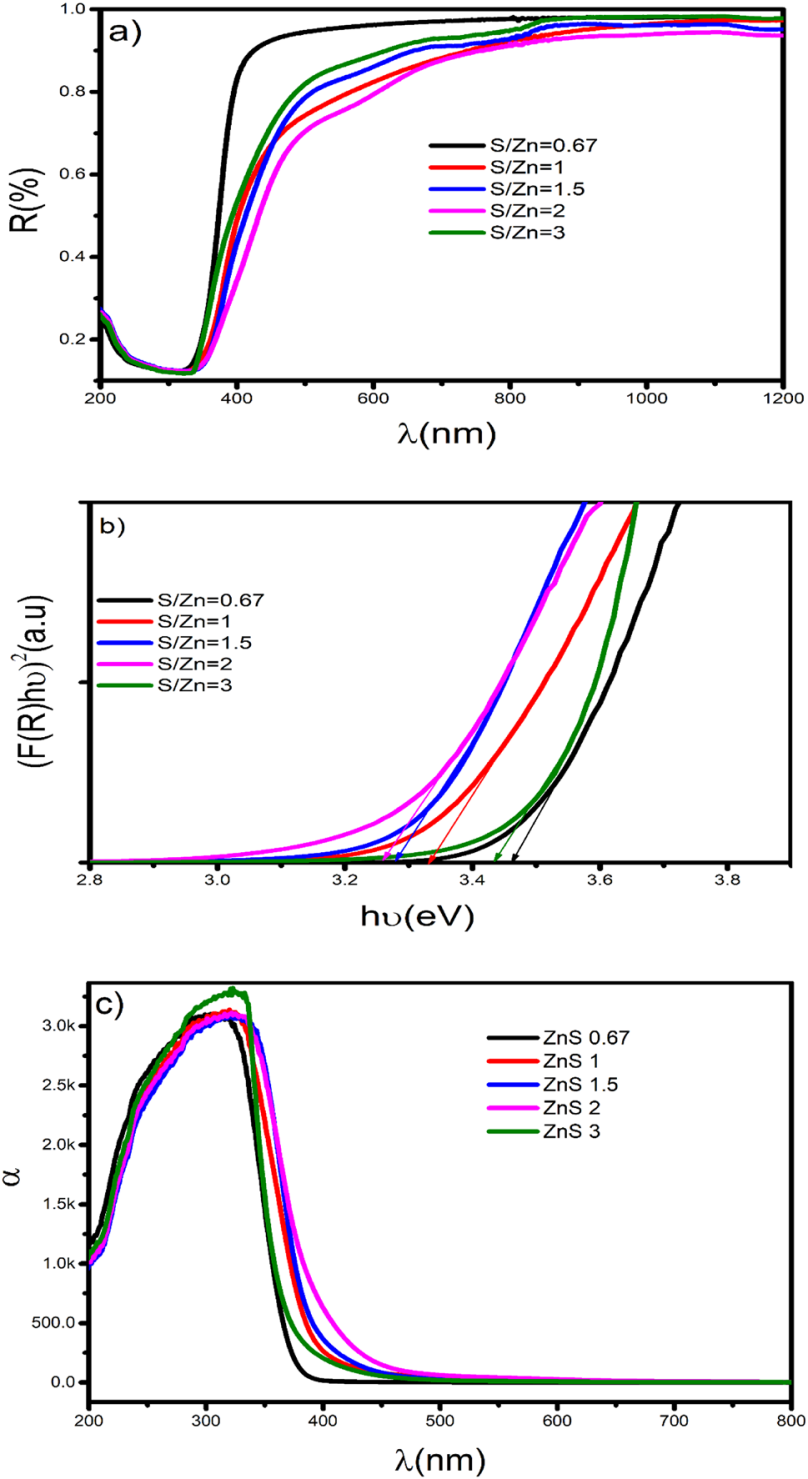
(**a**) Reflectance spectra of ZnS samples. (**b**) (F(R)h ν )^2^ Vs h ν plot (**c**) absorption spectra.

The prepared ZnS0.67, ZnS1, ZnS1.5, ZnS2, ZnS2.5, and ZnS3 samples' UV–Vis DRS are examined to determine the impact of the defects on the electronic and optical properties of ZnS NPs. The absorbance plot in Fig. 6c demonstrates that all prepared samples absorb sunlight, showing that all ZnS NPs have visible-light-induced PC activity resulting from the vacancy states in their band structures. Zn deficiency samples show high absorption intensity compared to other samples. The DR (Fig. 6a) shows a sharp hike in the reflection in the UV region, between 330 and 350 nm for different samples, corresponding to the transition from the valence to the conduction bands (CB) of the ZnS NPs. It should be also noted that reflection is very high in the wavelength region of 400 and 1200 nm for all samples.

The Kubelka–Munk function was utilized on the diffused reflectance (R) information to appraise the absorption of the sample^73,74^

11 $$\mathrm{F}(\mathrm{R}) =\frac{(1-\mathrm{ R}){)}^{2}}{2R}$$

The band gap (Eg) values can be assessed from Tauc relations (Fig. 6b) for direct band gap materials in the structure^75,76^,

12 $${(F(R) h \nu )}^{2}=\mathrm{A }(\mathrm{h \nu }-\mathrm{ Eg})$$

where 'A' is a constant and 'ν' is the frequency. The band gap values are determined from the extrapolation of the Kubelka–Munk function. The determined Eg for various examples are displayed in Table 3. The Eg varies from 3.28 to 3.49 eV for different ZnS samples. The Eg value is minimum for the ZnS2 sample and maximum for the ZnS3 sample.

#### Photoluminescence (PL)

**Figure 7:**
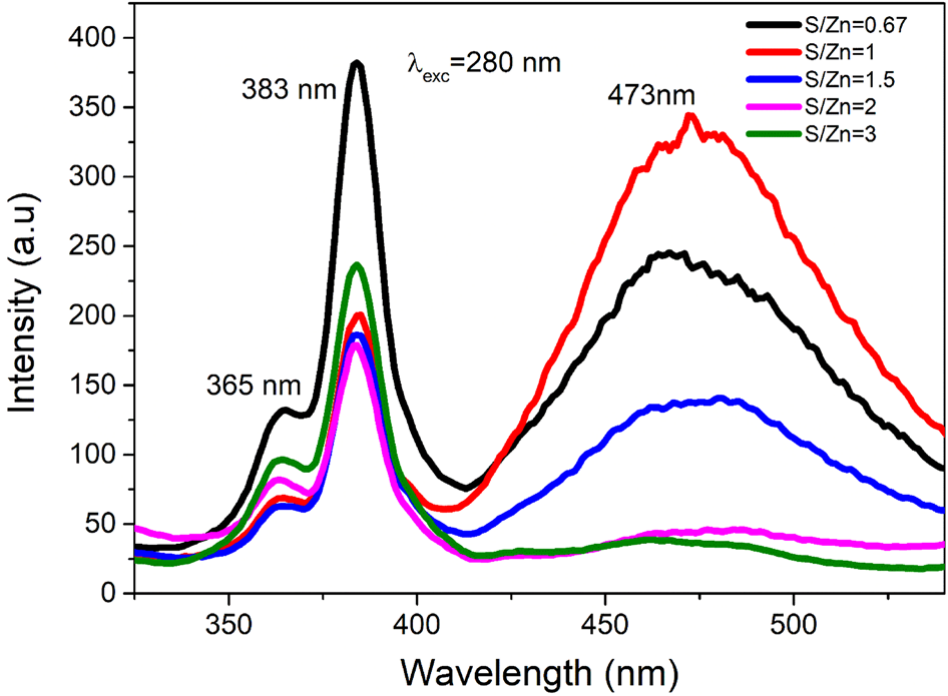
PL Spectra of ZnS nano structures.

**Figure 8:**
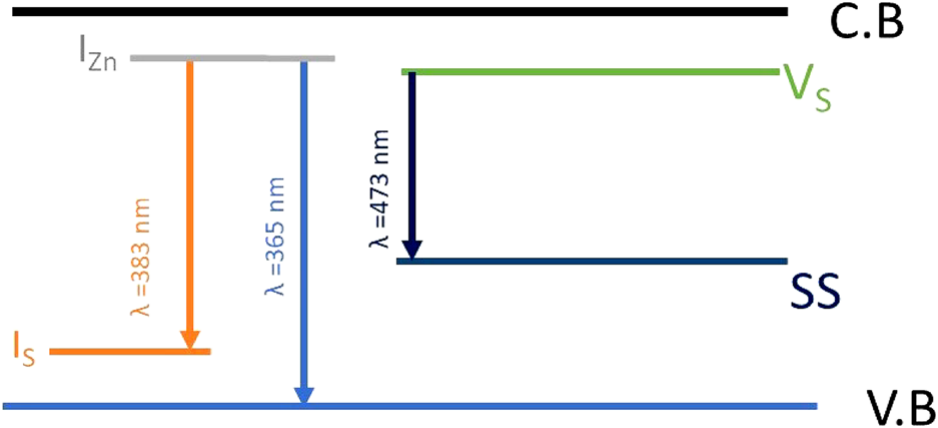
Energy band gap diagram.

Figure 7 shows the Photoluminescence spectra of ZnS nanoparticles with different S/Zn stoichiometric proportions for an exciting wavelength, 280 nm. The S/Zn ratio fluctuated from 0.67 to 3 in the precursor medium. According to the report by J. Zhou et al*.*, the point defects that act as luminous centers during photoluminescence processes are linked to defect emission^63^. In pure ZnS, different forms of point defects are commonly found as Sulphur vacancies (Vs), Zinc vacancies (V_Zn_), interstitial Sulphur atoms (I_S_), and interstitial Zinc atoms (I_Zn_)^37^. According to reports, the point defects that act as luminous sites during photoluminescence processes are linked to defect emission. The PL spectra show the emissions are at 365 nm, 383 nm, and 473 nm. The emission wavelengths around 365 nm and 383 nm have arisen due to the transition from interstitial Zn. Transitions corresponding to 365 nm are from I_Zn_ in the samples. The transition at 383 nm corresponds to the transition from I_Zn_ to I_S_ interstitial states. The same transition is observed by B. L. Devi et al., in their work^77^. The intensity of broad blue emission (473 nm) from the prepared ZnS NPs differed with changes in proportion. According to Lalitha Devi et al., the strong emission at 473 nm was caused by the recombination of holes at the surface state (SS) and electrons trapped by Vs sites. At ZnS0.67, the example shows solid blue discharge with the peak extreme at 473 nm. Notwithstanding, as the sulphide fixation is expanded, the intensity of the blue band diminishes impressively and nearly vanishes at an abundance S level for ZnS3. In a pure ZnS, the possible emission sites are related to surface or lattice deformities or local impurities with a low concentration of sulphide particles, during synthesis, the ZnS formed will have a bigger number of Vs, which can serve as doubly ionized donor places. Manzoor et al., and Lu et al., observed that similar variety in the intensities of all emission bands in undoped ZnS with change in stoichiometry suggests their relationship with Vs centers^78–80^. The energy level diagram (Fig. 8) depicts possible emission processes in ZnS nanoparticles. The crystal defect is responsible for these defect-induced emission peaks and the variations in PL intensity.

#### Photocatalytic (PC)

Defect engineering is a low-cost and effective modification technique for designing and developing single-phase catalysts for atomic-scale regulation and the formation of active sites on photocatalyst surfaces^81–83^. Of the various defects, surface vacancies can control the surface atomic architecture and local coordination structure of the catalyst, exposing and activating the surface atom to make a single-phase catalyst possess high activity. Furthermore, other than creating defect levels and increasing the photocatalyst's photoabsorption capability, the anion and cation vacancy defects have unique impacts^84^. Anion vacancies (S) have been shown to attract photogenerated electrons and limit carrier recombination^85,86^, whereas cation (Zn) vacancies can boost surface charge transfer and increase the valence band (VB) position to reduce h + oxidation capacity^37,87,88^. As a result, the vacancies enhance the photocatalytic activity and stability of the catalyst.

**Figure 9:**
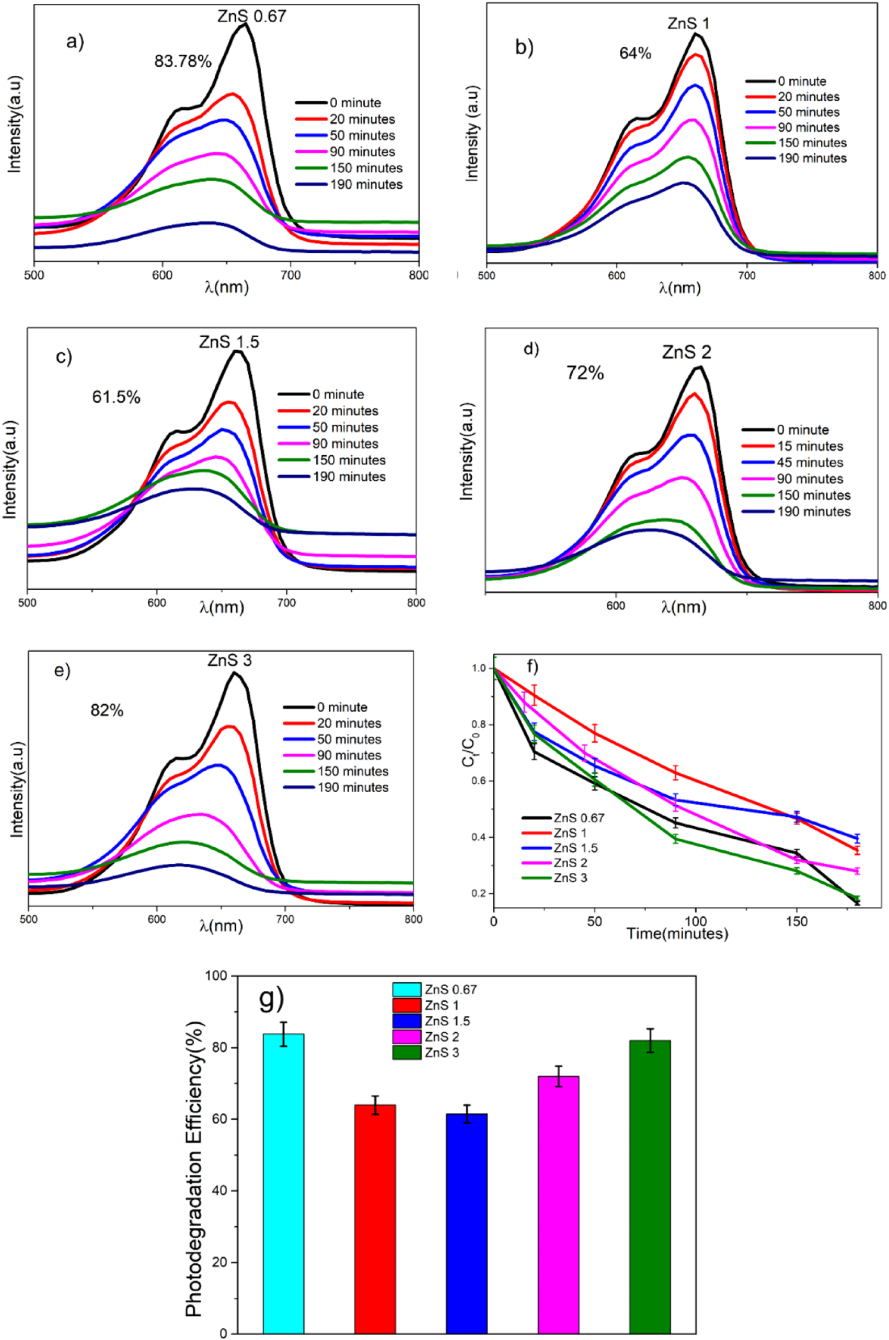
(**a**–**e**) time-dependent absorption spectra of photocatalytic degradation of MB dye solution with ZnS photocatalyst. (**f**) Photodegradation of MB Dye solution with ZnS photocatalyst. (**g**) Percentage photodegradation efficiency of ZnS photocatalyst.

The presence of defective states in the synthesized samples is confirmed by Raman spectra, XPS, and PL investigations, and hence, visible-light PC activities are critical to our investigations. Figure 9 depicts the MB solution's degradation at set intervals of sunshine exposures. The crystallinity, surface area, and shape are known to affect how the PC process operates, The PC activity may be enhanced by lowering the recombination of photogenerated electron–hole (e^−^–h^+^) pairs, stretching out the excitation wavelength to a smaller energy range and expanding surface-adsorbed reactant species^43,89,90^. In general, the system for photocatalysis starts when supra-band hole photons are explicitly absorbed in this manner, creating (e^−^–h^+^) pairs in the semiconductor particles. Following this, the charge carriers are dispersed across the molecules' surfaces, where they mix with water molecules to produce highly reactive forms of peroxide (O^2−^) and hydroxyl radical (OH), which cause the degradation of adsorbed molecules. The following diagram demonstrates how methylene blue is photo-catalytically degraded over a ZnS photocatalyst. Adsorption of the dye onto the outer layer of the ZnS nanostructure is the first step^65^. When dye-adsorbed ZnS nanostructures are exposed to sunlight, (e^−^–h^+^) pairs are formed.

$$ZnS+h\nu \to {e}^{-}\left(CB\right)+{h}^{+}(VB)$$

The light-induced electrons in the CB of ZnS associate with the oxygen atoms adsorbed on ZnS to shape superoxide anion extremists (${O}_{2}^{\cdot -}$^).^ The holes created in the valence band (VB) of ZnS react with surface hydroxyl groups to deliver extremely reactive (•OH) radicals^26^.

$${O}_{2}+{e}^{-}\to {O}_{2}^{\cdot -}$$

$${h}^{+}+{OH}^{-}\to \cdot OH$$

These holes $({h}^{+})$ can prompt the separation of water atoms in the fluid arrangement, creating radicals. The exceptionally reactive (⋅OH) radicals and superoxide radicals (O^2⋅−^) can react with methylene blue dye adsorbed on ZnS nanostructures and lead to its decomposition^39^.

$${h}^{+}+{H}_{2}O\to {H}^{+}+\cdot OH$$

The figure shows the time-dependent absorption spectra of PC degradation of MB dye under sunlight irradiation. The photodegradation is more for the samples having S/Zn = 0.67 and 3. Zhou et al., Hao et al., and Chen et al. also used the same strategy to improve the light-harvesting properties of photocatalyst^37,63,91^. The reason for the high PC degradation of the sample with S/Zn = 0.67 is S vacancies in the crystal. For ZnS, each S^2−^ ion forms a SZn_4_ tetrahedron with four Zn^2+^ ions around it, while each Zn^2+^ ion forms a ZnS_4_ tetrahedron with four S^2-^ ions surrounding it. All four Zn atoms around a S vacancy in a SZn_4_ tetrahedron transform into a ZnS_4_ pyramid with one sp^3^ dangling bond when one S atom is removed from the structure. When the composition surrounding the vacancy site is loosened, the symmetry is lowered, which causes the 1t level to split and a huge increase in the energy difference between the 1t and 1a levels. The result is that the three empty levels approach the CB minimum, while the 1a level approaches the VB maximum and becomes doubly filled. Around an S vacancy in the relaxed structure, one ZnS_3_ pyramid that becomes powerfully pyramidal in the loosened structure surrounding an S vacancy has its dangling bond double-filled to become a lone pair. The optical excitations connected to the filled defect level are then close to the VB maximum, while the unfilled defect levels below the CB minimum cause the ZnS samples with S vacancies to absorb visible light^71^. Additionally, these defect states capture photogenerated electrons and holes, which delay their recombination and improve the PC performance of ZnS samples with S vacancies^26,92^. Additionally, S and Zn vacancies could cause a little deviation of the perfect ZnS and lower its volume^93^. After geometric relaxations, the cell volume loss caused by S-vacancy is more noticeable than that caused by Zn-vacancy since it occupies a larger volume. S vacancies in ZnS crystals are more difficult to introduce than V_Zn_. The energy required to produce S vacancies (7.05 eV) is larger than that required to form V_Zn_ (5.99 eV). Therefore, more V_Zn_ is expected in ZnS crystals than in S vacancies. The V_Zn_ is the reason for the high degradation of sample with S/Zn = 3. V_Zn_ in the ZnS photocatalysts is responsible for the higher visible-light absorption and excellent charge separation efficiencies of ZnS catalysts, which result in better visible-light PC activities. The similar observations were reported by Hao et al*.*^37^. Zhang et al., also conducted visible light PC activity by varying the molar ratios. They reported maximum PC activity for ZnS with S/Zn = 2 prepared by solid-state reaction^89^. Fang et al*.,* adjusted the number of V_S_ and phase junctions to achieve optimal visible light activity on ZnS synthesized by hydrothermal and solvothermal methods^43^. Hao et al*.,* reported high visible light PC activity for ZnS synthesized by hydrothermal technique with Zn/S = 2.5^37^. Table 4 contains a few reported literatures on defect engineering and improved applications for comparison.

**Table 4:** Delineation of several synthesis techniques for defect creating and applications.

Material	Synthesis method	Defect created	Enhanced properties	Applications	References
ZnS	Chemical method	S vacancy, Zn vacancy	Green and orange luminescence	Photoelectrical applications	^20^
ZnS	Hydrothermal method	Zn vacancy	Charge separation and the electrons transfer are more efficient	Visible Photocatalytic hydrogen evolution	^37^
ZnS	Modified hydrothermal method	S vacancy	Photosensitization	Visible light photocatalysis	^43^
ZnS	One Pot Hydrothermal method	S vacancy, Zn vacancy	Charge separation efficiency	Visible light photocatalysis	^71^
BiOBr	Hydrothermal method	O vacancy	Enhanced charge separation	Photocatalytic N fixation	^96^
TiO_2_	Hydrothermal and photo-assisted reduction	Doped with transition metals	Electron–hole recombination time	Photocatalytic activity	^97^
FeOOH/rGO composites	Hummer's method	O vacancy	photogenerated electron–hole separation	Photo-fenton-like catalysts	^98^
MoS_2_	Hydrothermal	S vacancy	Increasing the active sites	Electro catalytic	^99^
BaSO_4_	Precipitation method	Ba vacancy	Improved adsorption	Photocatalytic removal of NO	^100,101^
ZnCdS/ZnS	Solvothermal method	Zn vacancy	Visible light absorption	Visible photocatalytic hydrogen evolution	^102^
g-C3N4	Hydrothermal	N vacancy	Visible light absorption	Visible light photocatalysis	^94^
ZrO_2_	Chemical method	O vacancy	Alteration of electronic structure	Photocatalytic activity	^103^

**Figure 10:**
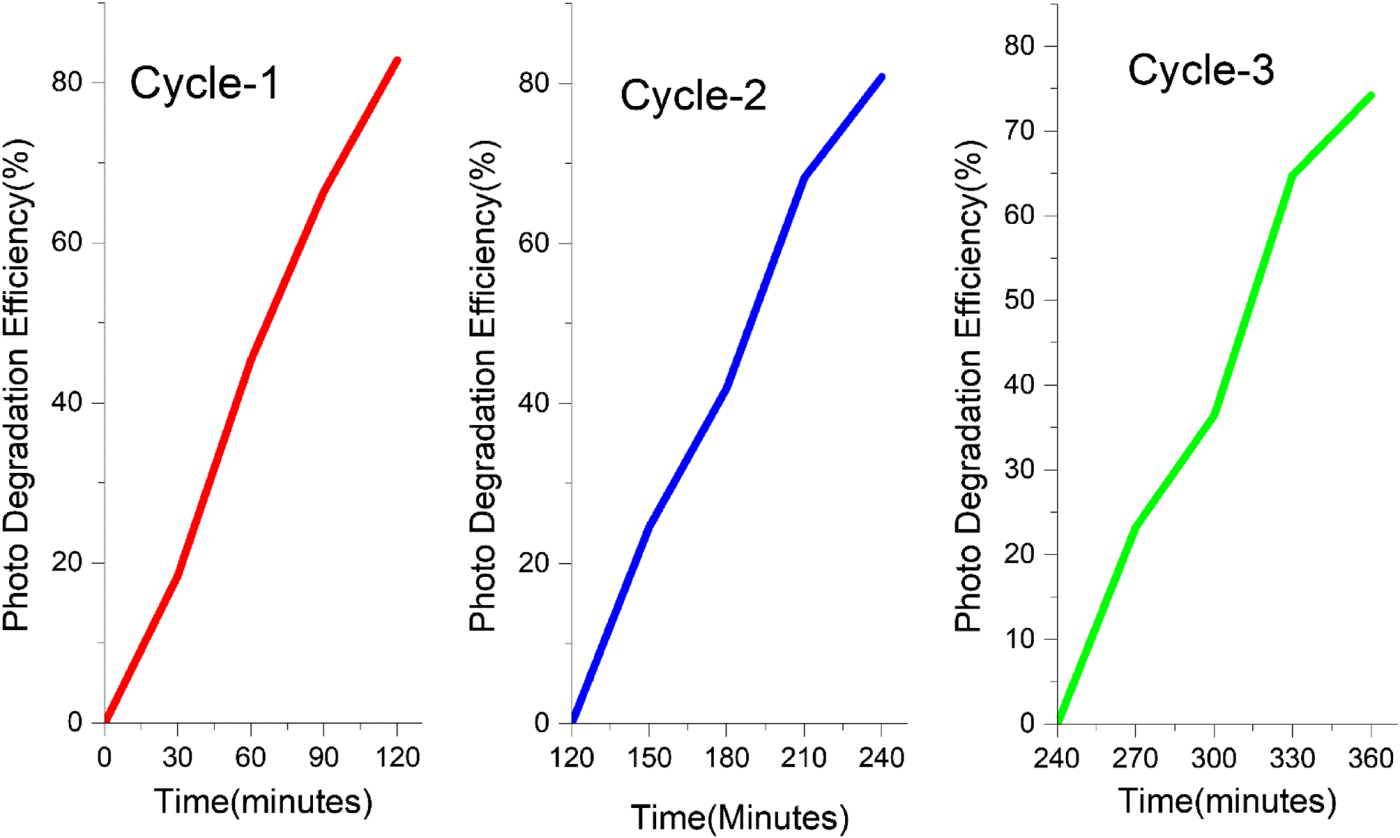
Recyclability tests of samples (three runs) involved with MB degradation.

Under identical settings, the recycling experiments were repeated three times to study the stability of the catalyst using the Xenon lamp^94^. The ZnS catalyst was extracted at the end of each cycle for recycling purposes using a centrifuge. Figure 10 depicts the results that were obtained after three cycles of testing. The dye degrades with no substantial changes in the rate of degradation up to the third cycle, which is a very important observation. However, a nominal decrease in decay percentage was observed, attributed to partial surface passivation by the decomposed MB dye^91^.

#### Brunauer–Emmett–Teller (BET)

BET techniques were used to determine the specific surface area of each sample. According to the experimental findings (Table 3), the Photodegradation Efficiency of the samples follows the order: ZnS0.67 > ZnS3 > ZnS2 > ZnS1 > ZnS1.5. However, the surface area follows a different trend: ZnS0.67 > ZnS1.5 > ZnS2 > ZnS3 > ZnS1. This disparity shows that defects within the materials have a greater impact on the visible-light photocatalysis of the defective ZnS systems^95^ than their surface area. The defects, like vacancies or defects created by the dopants, can provide energy levels in the band gap, making it easier to separate charges and absorb visible light, enhancing photocatalytic activity. These findings demonstrate how the defects can be used to tune the photocatalytic activity of ZnS materials under visible-light irradiation.

In the present work, we investigated at the manner in which different parameters including size, band gap energy, strain, surface area, and defects affected the photocatalytic activity of samples with varied S/Zn ratios. Interestingly, after analysing the data, we observed that several parameters, such as size, band gap energy, strain, and surface area, are known to affect the photocatalytic activity of the samples. However, parameters other than the above mentioned may also be impacting the photocatalytic performance of the present samples. The existence of defects in the samples appeared to play an important role in influencing their photocatalytic activity. Increased defect densities resulted in enhanced photocatalytic activity. It suggests that defects rather than size, band gap energy, strain, or surface area, may be the most important element impacting these samples' photocatalytic efficiency. These findings emphasize the relevance of incorporating defect engineering methodologies into photocatalytic material design and development. It may be feasible to increase the photocatalytic activity and overall performance of materials by deliberately introducing and controlling defects. In order to optimize the design of effective photocatalysts, further research is needed to understand the unique function of defects and their impact on photocatalytic mechanisms.

## Conclusion

ZnS NPs with different S/Zn molar ratio have been synthesized using a low-temperature hydrothermal route for photocatalysis applications. Through defect engineering, the Zn and S defects are successfully introduced into the ZnS crystal, which also alters the band structure of ZnS. The defects are generated on the surface during the synthesis, which is evident from RAMAN, XPS, and PL. The crystallinity and phase formation of the samples have been confirmed through XRD and Raman analysis. Through Scherrer, W–H model, and size strain plot, the impact of crystal defect on the structural characteristics of ZnS nanoparticles was studied. There is a strong effect of S/Zn molar concentration on the energy gap, and the band gap has been reduced from 3.49 to 3.28 eV (ΔΕ = 0.21 eV). The ICP-OES data reveal that all samples follow the predicted S/Zn ratio trend. The PL spectra show that the ZnS nanoparticles emit dual colors, which are violet and blue. The NPs agglomeration can be seen in TEM images; however, the size of synthesized NPs is in the quantum dots range. SEM study reveals the different morphology of the synthesized ZnS NPs. The vacancy-dependent visible light photocatalytic activity has been studied. The photocatalysis degradation activity was very high for Sulfur defect-rich sample ZnS0.67 and Zn defect-rich ZnS3 samples. Our study reveals that the developed NPs of ZnS with rich defects are stable photocatalysts and highly applicable for waste-water treatment.

## Data Availability

The data that support the findings of this study are available from [corresponding author], but restrictions apply to the availability of these data, which were used under license for the current study and so are not publicly available. Data are, however available from the authors upon reasonable request and with permission of [corresponding author].

## References

[1] Yu FP, Ou SL, Yao PC, Wu BR, Wuu DS (2014). Structural, surface morphology and optical properties of ZnS films by chemical bath deposition at various Zn/S molar ratios. J. Nanomater..

[2] Li ZQ, Shi JH, Liu QQ, Wang ZA, Sun Z, Huang SM (2010). Effect of [Zn]/[S] ratios on the properties of chemical bath deposited zinc sulfide thin films. Appl. Surf. Sci..

[3] Dedova T (2014). Effect of Zn: S Molar ratio in solution on the properties of ZnS thin films and the formation of ZnS nanorods by spray pyrolysis. Phys. Status Solidi Appl. Mater. Sci..

[4] Liu J (2009). Synthesis of ZnS nanoparticles via hydrothermal process assisted by microemulsion technique. J. Alloys Compd..

[5] Zhang YC, Wang GY, Hu XY, Chen WW (2006). Solvothermal synthesis of uniform hexagonal-phase ZnS nanorods using a single-source molecular precursor. Mater. Res. Bull..

[6] Vijayan S, Dash CS, Umadevi G, Sundararajan M, Mariappan R (2021). Investigation of structural, optical and antibacterial activity of ZnS nanoparticles. J. Clust. Sci..

[7] Desai NV, Shaikh IA, Raval AV, Raval KG, Shah DV (2021). Investigating the luminescent property of transition metal doped ZnS nanoparticles synthesised by co-precipitation method. IOP Conf. Ser. Mater. Sci. Eng..

[8] Markov, A. A., & Filimonov, I. A. Modeling of thermal radiation during zinc sulfide synthesis via combustion in a wet inert gas environment. In *AIP Conf. Proc.*, vol. 2181, no. November, 2019. 10.1063/1.5135672.

[9] Liu C, Wu X (2018). Reaction temperature-dependent growth of ZnS nanomaterials. Micro Nano Lett..

[10] Wang C, Hu B, Chen L, Liu N, Li J (2020). Preparation and characterization of ZnS nanostructures. Optik (Stuttg).

[11] Fang X (2009). Single-crystalline ZnS nanobelts as ultraviolet-light sensors. Adv. Mater..

[12] Fang X, Bando Y, Ye C, Shen G, Golberg D (2007). Shape- and size-controlled growth of ZnS nanostructures. J. Phys. Chem. C.

[13] Hikavyy A, Neyts K, Stuyven G, Poelman D, De Visschere P (2002). Photoluminescent ZnS: Cu phosphor films made with atomic-layer chemical vapor deposition and thermal evaporation. J. Soc. Inf. Disp..

[14] Su FH, Fang ZL, Ma BS, Ding K, Li GH, Xu SJ (2004). Temperature and pressure behavior of the emission bands from Mn-, Cu-, and Eu-doped ZnS nanocrystals. J. Appl. Phys..

[15] Anandan S (2007). Photocatalytic activity of La-doped ZnO for the degradation of monocrotophos in aqueous suspension. J. Mol. Catal. A Chem..

[16] Sridevi D, Rajendran KV (2010). Enhanced photoluminescence of ZnS nanoparticles doped with transition and rare earth metallic ions. Chalcogenide Lett..

[17] He Z, Su Y, Chen Y, Cai D, Jiang J, Chen L (2005). Self-catalytic growth and photoluminescence properties of ZnS nanostructures. Mater. Res. Bull..

[18] Becker WG, Bard AJ (1983). Photoluminescence and photoinduced oxygen adsorption of colloidal zinc sulfide dispersions. J. Phys. Chem..

[19] Biswas S, Kar S (2008). Fabrication of ZnS nanoparticles and nanorods with cubic and hexagonal crystal structures: A simple solvothermal approach. Nanotechnology.

[20] Wang X, Shi J, Feng Z, Li M, Li C (2011). Visible emission characteristics from different defects of ZnS nanocrystals. Phys. Chem. Chem. Phys..

[21] Wei Z (2018). Synthesis and luminescent modulation of ZnS crystallite by a hydrothermal method. ACS Omega.

[22] Deshpande BD, Agrawal PS, Yenkie MKN, Dhoble SJ (2020). Prospective of nanotechnology in degradation of waste water: A new challenges. Nano-Struct. Nano-Objects.

[23] Landrigan PJ (2018). The Lancet Commission on pollution and health. Lancet.

[24] Hermabessiere L (2017). Occurrence and effects of plastic additives on marine environments and organisms: A review. Chemosphere.

[25] Chowdhary, P., Bharagava, R. N., Mishra, S., & Khan, N. Role of industries in water scarcity and its adverse effects on environment and human health. In *Environmental Concerns and Sustainable Development* pp. 235–256 (Springer, Singapore, 2020).

[26] Lee GJ, Wu JJ (2017). Recent developments in ZnS photocatalysts from synthesis to photocatalytic applications—A review. Powder Technol..

[27] Li D, Shi W (2016). Recent developments in visible-light photocatalytic degradation of antibiotics. Chin. J. Catal..

[28] Ruan X (2019). Facile fabrication of Ag2O/Bi12GeO20 heterostructure with enhanced visible-light photocatalytic activity for the degradation of various antibiotics. J. Alloys Compd..

[29] Meng F (2012). Visible light photocatalytic activity of nitrogen-doped La2Ti2O7 nanosheets originating from band gap narrowing. Nano Res..

[30] Djurišić AB, He Y, Ng AMC (2020). Visible-light photocatalysts: Prospects and challenges. APL Mater..

[31] Khandelwal A, Maarisetty D, Baral SS (2022). Fundamentals and application of single-atom photocatalyst in sustainable energy and environmental applications. Renew. Sustain. Energy Rev..

[32] Sharma K (2021). ZnS-based quantum dots as photocatalysts for water purification. J. Water Process Eng..

[33] Liu C (2021). Research progress of defective MoS2 for photocatalytic hydrogen evolution. J. Korean Ceram. Soc..

[34] Wang Z, Xiao M, You J, Liu G, Wang L (2022). Defect engineering in photocatalysts and photoelectrodes: From small to big. Accounts Mater. Res..

[35] Maarisetty D, Baral SS (2021). Effect of defects on optical, electronic, and interface properties of NiO/SnO 2 heterostructures: Dual-functional solar photocatalytic H 2 production and RhB degradation. ACS Appl. Mater. Interfaces.

[36] Daskalakis I (2020). Surface defect engineering of mesoporous Cu/ZnS nanocrystal-linked networks for improved visible-light photocatalytic hydrogen production. Inorg. Chem. Front..

[37] Hao X, Wang Y, Zhou J, Cui Z, Wang Y, Zou Z (2018). Zinc vacancy-promoted photocatalytic activity and photostability of ZnS for efficient visible-light-driven hydrogen evolution. Appl. Catal. B Environ..

[38] Arai T (2008). Cu-doped ZnS hollow particle with high activity for hydrogen generation from alkaline sulfide solution under visible light. Chem. Mater..

[39] Jothibas M, Manoharan C, Jeyakumar SJ, Praveen P, Punithavathy IK, Richard JP (2018). Synthesis and enhanced photocatalytic property of Ni doped ZnS nanoparticles. Sol. Energy.

[40] Wang Y, Wu J, Zheng J, Xu R (2011). Highly active ZnxCd1-xS photocatalysts containing earth abundant elements only for H2 production from water under visible light. Catal. Sci. Technol..

[41] Sun, H., Zhao, X., Zhang, L., & Fan, W. Origin of the enhanced visible photocatalytic activity in (N, C )-codoped ZnS studied from density functional theory, pp. 2218–2227 (2011).

[42] Zhou Y, Chen G, Yu Y, Feng Y, Zheng Y, He F, Hana Z (2014). An efficient method to enhance the stability of sulphide semiconductor photocatalysts: A case study of N-doped ZnS. Phys. Chem..

[43] Fang Z (2015). Defect engineering and phase junction architecture of wide-bandgap ZnS for conflicting visible light activity in photocatalytic H2 evolution. ACS Appl. Mater. Interfaces.

[44] Iranmanesh P, Saeednia S, Nourzpoor M (2015). Characterization of ZnS nanoparticles synthesized by co-precipitation method. Chin. Phys. B.

[45] Jubeer EM, Manthrammel MA, Shkir M, Subha PA, Yahia IS, Alfaify A (2021). Optik Microwave assisted synthesis of quantum dots like ZnS nanoparticles for optoelectronic applications: An effect of CTAB concentrations. Optik (Stuttg)..

[46] Quynh Hoa TT, Van Vu L, Canh TD, Long NN (2009). Preparation of ZnS nanoparticles by hydrothermal method.. J. Phys. Conf. Ser.

[47] Zhou X, Yang Q, Wang H, Huang F, Zhang J, Xu S (2019). Influences of reaction temperature, holding time and S/Zn molar ratio on structure, morphology, optical and electrical properties of ZnS nanoparticles synthesized by hydrothermal method. J. Mater. Sci. Mater. Electron..

[48] Thambidurai M, Murugan N, Muthukumarasamy N, Agilan S, Vasantha S, Balasundaraprabhu R (2010). Influence of the Cd/S molar ratio on the optical and structural properties of nanocrystalline CdS thin films. J. Mater. Sci. Technol..

[49] Bindu P, Thomas S (2014). Estimation of lattice strain in ZnO nanoparticles: X-ray peak profile analysis. J. Theor. Appl. Phys..

[50] Arandhara G, Bora J, Saikia PKK (2020). Effect of pH on the crystallite size, elastic properties and morphology of nanostructured ZnS thin films prepared by chemical bath deposition technique. Mater. Chem. Phys..

[51] Alqahtani A, Husain S, Somvanshi A, Khan W (2019). Structural, morphological, thermal and optical investigations on Mn doped GdCrO3. J. Alloys Compd..

[52] Mote VD, Purushotham Y, Dole BN (2012). Williamson-Hall analysis in estimation of lattice strain in nanometer-sized ZnO particles. J. Theor. Appl. Phys..

[53] Suganthi N, Pushpanathan K (2018). Photocatalytic degradation and ferromagnetism in mesoporous La doped ZnS nanoparticles. J. Mater. Sci. Mater. Electron..

[54] Aly KA, Khalil NM, Algamal Y, Saleem QMA (2016). Lattice strain estimation for CoAl2O4 nano particles using Williamson-Hall analysis. J. Alloys Compd..

[55] Nawaz, M. A., *et al.* Microstructural study of as grown and 650 °C annealed ZnO nanorods: X-ray peak profile analysis. **11**(2), 537–546 (2016).

[56] Maniammal K, Madhu G, Biju V (2017). X-ray diffraction line profile analysis of nanostructured nickel oxide: Shape factor and convolution of crystallite size and microstrain contributions. Phys. E Low-Dimens. Syst. Nanostruct..

[57] Xin M, Liao LM, Han F (2021). Optical properties of ZnS: Ce nanocrystals prepared by hydrothermal method. J. Lumin..

[58] Madhavi J (2019). Comparison of average crystallite size by X-ray peak broadening and Williamson-Hall and size–strain plots for VO2+ doped ZnS/CdS composite nanopowder. SN Appl. Sci..

[59] Thandavan TMK, Gani SMA, Wong CS, Nor RM (2015). Evaluation of Williamson-hall strain and stress distribution in ZnO nanowires prepared using aliphatic alcohol. J. Nondestruct. Eval..

[60] Khorsand Zak A, Abd WH, Majid ME (2011). X-ray analysis of ZnO nanoparticles by Williamson-Hall and size–strain plot methods. Solid State Sci..

[61] Riazian M, Yousefpoor M (2020). Photocatalytic activity, nanostructure and optical properties of 3D ZnS urchin-like via hydrothermal method. Int. J. Smart Nano Mater..

[62] Cheng YC (2009). Raman scattering study of zinc blende and wurtzite ZnS. J. Appl. Phys..

[63] Zhou J, Zhao J, Liu R (2020). Defect engineering of zeolite imidazole framework derived ZnS nanosheets towards enhanced visible light driven photocatalytic hydrogen production. Appl. Catal. B Environ..

[64] Nilsen WG (1969). Raman spectrum of cubic ZnS. Phys. Rev..

[65] Ye Z, Kong L, Chen F, Chen Z, Lin Y, Liu C (2018). A comparative study of photocatalytic activity of ZnS photocatalyst for degradation of various dyes. Optik (Stuttg).

[66] Dhara, S., *et al.* Deformation potential dominated phonons in ZnS quantum dots. pp. 1–12.

[67] Serrano J (2004). Raman scattering in β-ZnS. Phys. Rev. B Condens. Mater. Phys..

[68] El-Desoky MM, El-Barbary GA, El Refaey DE, El-Tantawy F (2018). Optical constants and dispersion parameters of La-doped ZnS nanocrystalline films prepared by sol–gel technique. Optik (Stuttg).

[69] Saikia D, Borah JP, Jangra M, Puzari A (2016). Investigation of photophysical properties of ZnS:Mn2+ nanoparticles. Indian J. Phys..

[70] Morrison C, Sun H, Yao Y, Loomis RA, Buhro WE (2020). Methods for the ICP-OES analysis of semiconductor materials. Chem. Mater..

[71] Yang X (2021). A straightforward one-pot approach to two new defect energy levels in ZnS. CrystEngComm.

[72] Goudarzi A (2009). Low-temperature growth of nanocrystalline Mn-doped ZnS thin films prepared by chemical bath deposition and optical properties. Chem. Mater..

[73] Shobana M, Meher SR (2019). E ff ect of cobalt doping on the structural, optical and magnetic properties of sol-gel derived ZnS nanocrystalline thin fi lms and ab initio studies. Thin Solid Films.

[74] Shahi AK, Pandey BK, Singh BP, Gupta BK, Singh S, Gopal R (2017). Photo physical studies of PVP arrested ZnS quantum dots. Electron. Mater. Lett..

[75] Aydin H, Aydin C, Al-Ghamdi AA, Farooq WA, Yakuphanoglu F (2016). Refractive index dispersion properties of Cr-doped ZnO thin films by sol-gel spin coating method. Optik (Stuttg).

[76] Ilican S, Caglar Y, Caglar M, Yakuphanoglu F (2009). The effects of substrate temperature on refractive index dispersion and optical constants of CdZn(S0.8Se0.2)2 alloy thin films. J. Alloys Compd..

[77] Devi BL, Rao KM, Kekuda D, Ramananda D (2018). Evolution of defects and their effect on photoluminescence and conducting properties of green-synthesized ZnS nanoparticles. Appl. Phys. A Mater. Sci. Process..

[78] Manzoor K, Vadera SR, Kumar N, Kutty TRN (2003). Synthesis and photoluminescent properties of ZnS nanocrystals doped with copper and halogen. Mater. Chem. Phys..

[79] Lu HY, Chu SY, Tan SS (2004). The characteristics of low-temperature-synthesized ZnS and ZnO nanoparticles. J. Cryst. Growth.

[80] Fang X (2011). ZnS nanostructures: From synthesis to applications. Prog. Mater. Sci..

[81] Wang M (2020). A hydrogen-deficient nickel-cobalt double hydroxide for photocatalytic overall water splitting. Angew. Chemie Int. Ed..

[82] Li H, Li J, Ai Z, Jia F, Zhang L (2018). Oxygen vacancy-mediated photocatalysis of BiOCl: Reactivity, selectivity, and perspectives. Angew. Chemie Int. Ed..

[83] Xue D, Xia H, Yan W, Zhang J, Mu S (2021). Defect engineering on carbon-based catalysts for electrocatalytic CO2 reduction. Nano-Micro Lett..

[84] Xiao B (2021). Synergistic effect of the surface vacancy defects for promoting photocatalytic stability and activity of ZnS nanoparticles. ACS Catal..

[85] Du C, Zhang Q, Lin Z, Yan B, Xia C, Yang G (2019). Half-unit-cell ZnIn2S4 monolayer with sulfur vacancies for photocatalytic hydrogen evolution. Appl. Catal. B Environ..

[86] Lee J, Ham S, Choi D, Jang D-J (2018). Facile fabrication of porous ZnS nanostructures with a controlled amount of S vacancies for enhanced photocatalytic performances. Nanoscale.

[87] Hao X (2018). Architecture of high efficient zinc vacancy mediated Z-scheme photocatalyst from metal-organic frameworks. Nano Energy.

[88] Mahvelati-Shamsabadi T, Goharshadi EK (2017). Photostability and visible-light-driven photoactivity enhancement of hierarchical ZnS nanoparticles: The role of embedment of stable defect sites on the catalyst surface with the assistant of ultrasonic waves. Ultrason. Sonochem..

[89] Zhang H, Chen X, Li Z, Kou J, Yu T, Zou Z (2007). Preparation of sensitized ZnS and its photocatalytic activity under visible light irradiation. J. Phys. D. Appl. Phys..

[90] Wang Z (2014). Progress on extending the light absorption spectra of photocatalysts. Phys. Chem. Chem. Phys..

[91] Juine RN, Sahu BK, Das A (2021). Recyclable ZnS QDs as an efficient photocatalyst for dye degradation under the UV and visible light. New J. Chem..

[92] Wang G (2015). Synthesis and characterization of ZnS with controlled amount of S vacancies for photocatalytic H2 production under visible light. Sci. Rep..

[93] Chen F, Cao Y, Jia D (2015). Facile synthesis of ZnS nanoparticles and their excellent photocatalytic performance. Ceram. Int..

[94] Hong Z, Shen B, Chen Y, Lin B, Gao B (2013). Enhancement of photocatalytic H2 evolution over nitrogen-deficient graphitic carbon nitride. J. Mater. Chem. A.

[95] Fang Z (2015). Defect engineering and phase junction architecture of wide-bandgap ZnS for conflicting visible light activity in photocatalytic H 2 evolution. ACS Appl. Mater. Interfaces.

[96] Xue X (2018). Oxygen vacancy engineering promoted photocatalytic ammonia synthesis on ultrathin two-dimensional bismuth oxybromide nanosheets. Nano Lett..

[97] Wu C-G, Chao C-C, Kuo F-T (2004). Enhancement of the photo catalytic performance of TiO2 catalysts via transition metal modification. Catal. Today.

[98] Wu X (2022). Engineering controllable oxygen vacancy defects in iron hydroxide oxide immobilized on reduced graphene oxide for boosting visible light-driven photo-Fenton-like oxidation. J. Colloid Interface Sci..

[99] Li L (2019). Role of sulfur vacancies and undercoordinated Mo regions in MoS 2 nanosheets toward the evolution of hydrogen. ACS Nano.

[100] Cui W (2019). Ba-vacancy induces semiconductor-like photocatalysis on insulator BaSO4. Appl. Catal. B Environ..

[101] Maarisetty D, Baral SS (2020). Defect engineering in photocatalysis: formation, chemistry, optoelectronics, and interface studies. J. Mater. Chem. A.

[102] Hao X, Xiang D, Jin Z (2021). Zn-vacancy engineered S-scheme ZnCdS/ZnS photocatalyst for highly efficient photocatalytic H 2 evolution. ChemCatChem.

[103] Kim HS (2022). Verifying the relationships of defect site and enhanced photocatalytic properties of modified ZrO2 nanoparticles evaluated by in-situ spectroscopy and STEM-EELS. Sci. Rep..

